# Stress urinary incontinence after male to female gender reassignment surgery: Successful use of a pubo-vaginal sling

**DOI:** 10.4103/0970-1591.33731

**Published:** 2007

**Authors:** Pankaj P. Dangle, Simon C. W. Harrison

**Affiliations:** Department of Urology, Orchard House, Aberford Road, Wakefield WF1 4DG, United Kingdom; Department of Urology, Pinderfields General Hospital, Orchard House, Aberford Road, Wakefield WF1 4DG, United Kingdom

**Keywords:** Gender reassignment, pubovaginal sling, urinary contienence

## Abstract

The case of a 50-year-old patient who had undergone male to female gender reassignment surgery is presented. She presented with mixed incontinence with symptoms of stress incontinence predominating. Initial conservative treatment was unsuccessful and subsequent videourodynamic assessment demonstrated urodynamic stress incontinence in association with a partially open bladder neck at rest. Also noted during the study was cough-induced detrusor overactivity. The option of inserting a pubo-vaginal sling using autologous rectus sheath was chosen. The procedure proved to be straightforward to perform and was uncomplicated. Subsequent follow-up demonstrated a resolution of her stress incontinence.

## INTRODUCTION

As we are aware, the management of stress urinary incontinence is well standardized in females with the use of pubovaginal sling and now with the less invasive approach of TVT and TOTVTs. In our case TVT or TOT was not used as we were unsure as to whether the neo-vaginal skin would heal over the sling.

The use of pubovaginal sling following gender assignment has not been reported previously. A case report on the use of intraurethral collagen injection suggests some degree of improvement in the incontinence.[1]

Use of artificial urinary sphincter is well known in cases of incontinence but literature search lacks any reports on its use in gender assigned patients.

## CASE REPORT

A 50-year-old patient, who had undergone male to female gender reassignment surgery nine years previously, was referred with a four-year history of urinary incontinence. During the first five years after surgery she had been free of urinary symptoms although in the early postoperative period she had developed a meatal stenosis which was treated by dilatation and meatoplasty. In addition, during that procedure, residual erectile tissue was excised from the area of the urethral meatus.

Urinary incontinence was of gradual onset and had been present for four years at the time of referral. Incontinence was noted to occur on coughing and movement but could also occur in association with a sense of urgency or with minimal sensation. She had two hourly day time frequency and nocturia once every night. She also complained of reduction in stream and terminal dribble. Pads were used to contain the urinary incontinence. She was taking oral estrogens in order to promote feminization.

On examination there was evidence of a prolapse of the posterior wall of the neo-vagina while the prostatic remnant was small and atrophic.

Investigations included a midstream urine sample and ultrasound of the urinary tract; these were normal.

**Figure 1:**
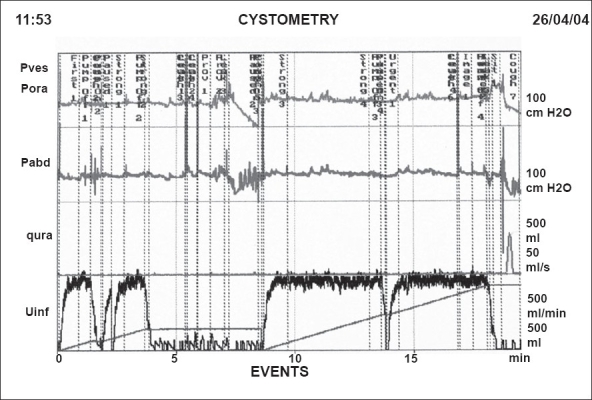
Preoperative urodynamic study showing cough induced detrusor overactivity with stress incontinence

Initial management was conservative with a regime of bladder and pelvic floor training along with the use of anticholinergic medication. The response to these measures was disappointing and hence further assessment with videourodynamic investigation was undertaken. This study showed normal bladder compliance but the bladder neck was partially open at rest. Stress incontinence was observed at 100 ml bladder capacity; at 400ml and abdominal leak point pressure of 70 cm of water was observed with evidence of gross urinary leakage. Cough-induced detrusor overactivity was also observed during the study. Voiding took place with complete bladder emptying and a maximum flow rate of 27 ml/sec with a detrusor pressure at maximum flow of 41 cm of H_2_O [Figure 1].

The patient wished to consider surgical treatment for her incontinence and the options of artificial sphincter, pubovaginal sling and urethral bulking agents were considered. A decision was made to undertake placement of a rectus sheath pubovaginal sling. Cystoscopy and examination under anesthesia showed an intact distal urethral sphincter which was situated within 2 cm of the external urethral meatus. The bladder neck was slightly open. The prostate was noted to be freely mobile which was taken to indicate that the puboprostatic ligaments and endopelvic fascia had become stretched or disrupted. A 1.5 × 15 cm sling was harvested from the rectus sheath and an inverted U incision made on the anterior wall of the neo-vagina. Pelvic dissection was carried out as a neo-vagina was created by dissection between the prostate and rectum. The sheath was secured just distal to the apex of the prostate. Lateral and cephalad dissection into the retropubic space proceeded using an identical technique to that used in a standard pubovaginal sling procedure in a female patient.[2] The sling was secured using 0 prolene sutures which were tied without tension over the rectus sheath. An uneventful postoperative recovery followed. The posterior wall prolapse was asymptomatic and therefore was not addressed. It would, however, seem reasonable to use a TVT or TOT in any future cases given that sling placement does not seem to be unduly difficult.

On subsequent follow-up, there was no complaint of stress incontinence although very occasional urge incontinence was reported. Her follow-up flow rate showed good emptying with very minimal residual urine. Patient declined pad testing and urodynamic study as she was completely dry.

## DISCUSSION

This is believed to be the first reported case of the use of a rectus sheath pubovaginal sling in the management of stress urinary incontinence in a male to female gender reassignment patient. Previous reports describe the use of periurethral collagen injections in this context.[1]

Stress urinary incontinence in the male is rare and essentially confined to patients who have undergone prostatic surgery or who have neuropathic disorders of the lower urinary tract. In the gender reassignment patient, the etiology of sphincter weakness might include iatrogenic damage to the external urethral sphincter or its nerve supply.[3,4]

A retrospective study from Belgium used questionnaires regarding voiding habits and lower urinary tract symptoms. They had 31 MTF and 92 FTM transsexuals with the incontinence rate of 19.3%in MTF and 50% in FTM. Of the six MTF transsexuals who suffered incontinence one had dribbling, two urge incontinence, two stress and one had mixed incontinence. The authors in the study have put forth different hypotheses for the lower urinary tract symptoms, which most likely are the effect of surgery. During the surgery for vaginoplasty a pocket is created between the bladder and the rectum that will contain the neo-vagina. This pocket is created by blunt dissection behind the bladder and prostate. The sphincter complex and the pelvic floor muscles are in the dissected area, so some of the observed stress incontinence could be attributed to the surgery. Also damage to nerves supplying the bladder and the change of position of the bladder itself could lead to incontinence.[3]

Another study from Switzerland aimed to determine if transsexuals have an increased risk of micturition disorders and if so which. In their study they had 25 patients of which 18 were MTF and seven were FTM transsexuals. In MTF transsexuals, a diverted stream, overactive bladder and stress urinary incontinence was a common problem. The authors assumed that the reasons for the development of incontinence might be surgery including pudendal nerve damage, hormonal reasons and ageing.[4]

It is also possible that there is a failure of effective pressure transmission to the prostatic and membranous urethra as a result of the failure of the fascial supports to the bladder neck and prostate. In this case the prostate was surprisingly mobile suggesting a possible hypermobility effect while the low abdominal leak point pressure is suggestive of the intrinsic weakness of the external sphincter. Some denervation of the external sphincter may have occurred as a result of the construction of the neo-vagina. The experience with this patient suggests that the use of a pubovaginal sling is both logical and applicable to this unusual clinical situation.

The autologous pubovaginal sling is a viable option for management of stress urinary incontinence in male to female gender reassigned patients.
